# Techno-economic analysis of the industrial production of a low-cost enzyme using *E. coli*: the case of recombinant β-glucosidase

**DOI:** 10.1186/s13068-018-1077-0

**Published:** 2018-03-29

**Authors:** Rafael da Gama Ferreira, Adriano Rodrigues Azzoni, Sindelia Freitas

**Affiliations:** 1 Departamento de Engenharia Química, Escola Politécnica, Universidade de São Paulo, São Paulo, SP Brazil; 2 Laboratório de Ciência e Tecnologia do Bioetanol, Centro Nacional de Pesquisa em Energia e Materiais, Campinas, SP Brazil; 3 Faculdade de Engenharia Química, Universidade Estadual de Campinas, Campinas, SP Brazil

**Keywords:** Industrial enzymes, Recombinant *E. coli*, Techno-economic analysis, Cellulases, β-Glucosidase, Process simulation

## Abstract

**Background:**

The enzymatic conversion of lignocellulosic biomass into fermentable sugars is a promising approach for producing renewable fuels and chemicals. However, the cost and efficiency of the fungal enzyme cocktails that are normally employed in these processes remain a significant bottleneck. A potential route to increase hydrolysis yields and thereby reduce the hydrolysis costs would be to supplement the fungal enzymes with their lacking enzymatic activities, such as β-glucosidase. In this context, it is not clear from the literature whether recombinant *E. coli* could be a cost-effective platform for the production of some of these low-value enzymes, especially in the case of on-site production. Here, we present a conceptual design and techno-economic evaluation of the production of a low-cost industrial enzyme using recombinant *E. coli*.

**Results:**

In a simulated baseline scenario for β-glucosidase demand in a hypothetical second-generation ethanol (2G) plant in Brazil, we found that the production cost (316 US$/kg) was higher than what is commonly assumed in the literature for fungal enzymes, owing especially to the facility-dependent costs (45%) and to consumables (23%) and raw materials (25%). Sensitivity analyses of process scale, inoculation volume, and volumetric productivity indicated that optimized conditions may promote a dramatic reduction in enzyme cost and also revealed the most relevant factors affecting production costs.

**Conclusions:**

Despite the considerable technical and economic uncertainties that surround 2G ethanol and the large-scale production of low-cost recombinant enzymes, this work sheds light on some relevant questions and supports future studies in this field. In particular, we conclude that process optimization, on many fronts, may strongly reduce the costs of *E. coli* recombinant enzymes, in the context of tailor-made enzymatic cocktails for 2G ethanol production.

**Electronic supplementary material:**

The online version of this article (10.1186/s13068-018-1077-0) contains supplementary material, which is available to authorized users.

## Background

Although the production of bulk chemicals and biofuels based on the enzymatic deconstruction of lignocellulosic biomass has been studied intensively over the last two decades, few studies have explicitly analyzed the production cost of the enzymes involved. Studies that did delve into the enzyme cost have focused on the economics of the cellulase mixture produced by the filamentous fungus *Trichoderma reesei* [1–5], which is generally considered to be the most efficient producer of cellulases and, accordingly, is currently the most prevalent producer of cellulases used in the industry [6, 7]. Nevertheless, the estimated cost of these fungal cellulases remains an important bottleneck in the manufacture of low-value-added products such as second-generation ethanol from lignocellulosic materials [3, 7–9]. However, it is widely known that the composition of the enzymatic cocktail secreted by *T. reesei* is not optimal for the industrial degradation of cellulosic biomass in terms of cellulase activity, notably owing to the low β-glucosidase (BGL) activity of this enzymatic cocktail [10, 11]. Therefore, supplementing the fungal cocktail with the enzymes whose activities the cocktail is lacking in is a potential method for process optimization, assuming that the increase in hydrolysis yield outweighs the cost of the supplementary enzymes. Moreover, producing these supplementary enzymes on-site may be more economical than doing so off-site because the former avoids transportation and formulation costs [2, 12]. However, to our knowledge, no techno-economic analysis of such an enzyme, particularly one produced on-site, has been carried out to date. In fact, there are surprisingly few techno-economic analyses of the microbial processes used to produce low- or intermediate-value-added proteins other than fungal cellulases. Furthermore, considering that approximately 90% of all industrial enzymes are produced by recombinant organisms [13], there are very few analyses of protein production by recombinant microorganisms, especially in the case of high-volume, low-value enzymes. Great efforts have been made in the last decade, particularly in Brazil, to make the industrial production of 2G ethanol via enzymatic hydrolysis of sugarcane biomass economically feasible [14–16].

*E. coli* is the most common host for the recombinant expression of cellulases and is one of the most common bacteria used to produce recombinant proteins in general [17–20]. The main advantages of using *E. coli* are well known, such as the ability of *E. coli* to grow rapidly on simple and inexpensive media, the ability of this bacteria to grow to high cell densities and achieve high levels of protein expression, the availability of strains with low proteolytic activities, and the large body of scientific knowledge concerning the physiology, genetics, and manipulation *of E. coli* [18, 21–24]. However, *E. coli* also presents disadvantages with respect to recombinant protein production: normally, the protein is not secreted by the cell, which often makes downstream processing more complex and expensive; the recombinant protein may aggregate and form insoluble particles called inclusion bodies, in which the protein is inactive; disulfide bonds may not form as intended, leading to protein denaturation and often to the formation of inclusion bodies; the cell machinery may stall or truncate proteins owing to codon bias, leading to low expression or loss of function; *E. coli* is not capable of performing post-translational modifications such as glycosylation; and the lipopolysaccharides that constitute the outer membrane of *E. coli* elicit strong immune responses in humans and other mammals [17–21]. The last four issues are usually more relevant when producing eukaryotic proteins for human or animal use, but these issues do not preclude *E. coli* from accounting for one-third of the recombinant proteins approved by the FDA for therapeutic purposes [23, 25].

Considering the aforementioned advantages, disadvantages, and knowledge gaps associated with *E. coli* recombinant protein processes, the aim of this work was to model, simulate, and economically assess the production in *E. coli* of a low-value-added recombinant protein, β-glucosidase, to be used as a supplementary enzyme in lignocellulose hydrolysis. Process parameters that are known to be significant, such as the process scale, biomass productivity, and recombinant protein-specific productivity, were evaluated. Furthermore, process characteristics that are rarely emphasized in the literature, such as the seed train expansion factor, bioreactor material, and cost contributions of the inducer and antibiotic compounds, were also examined, thereby contributing to a better understanding of the factors that affect the technical and economic feasibility of such processes.

## Methods

### Design basis

The production scale was based on the assumption that enzyme manufacturing would be integrated with a sugarcane-based 1G + 2G ethanol plant (that is, on-site), using a 100 m^3^ bioreactor. With respect to product specifications, it was assumed that the enzyme should be stabilized in a citrate buffer of pH 5.8 and concentrated to a titer of 15 g/L. The main parameters used for the design are summarized in Table 1.

**Table 1:** Main parameters used for the design of the recombinant enzyme production process

Parameter	Assumption
Enzyme titer after primary recovery and concentration	15 g/L
Annual operating time of the enzyme production unit	7920 h (330 days)
Nominal volume of the main bioreactor	100 m^3^
Maximum working volume of the fermenter	80%
Temperature	26 °C
Overpressure	150 kPa
Fermenter material	Stainless steel—grade 316 (SS316)
µ	0.23 h^−1^
pH	6.8
Glucose concentration (during fed-batch phase)	1.5 g/L
pO_2_	20%
Nominal volume of the main bioreactor	100 m^3^
Enzyme titer after primary recovery and concentration	15 g/L
Annual operating time of the enzyme production unit	7920 h (330 days)

**Fig. 1:**
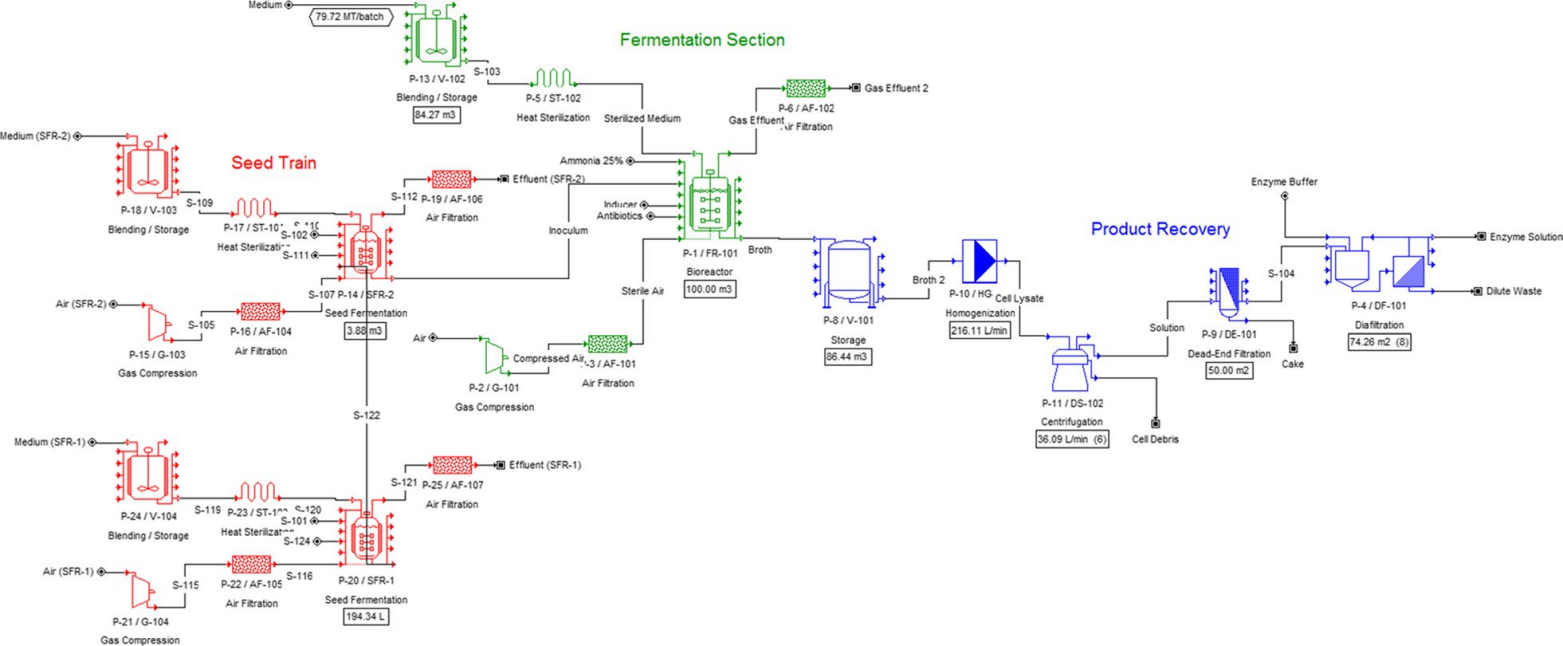
Flowsheet of the proposed recombinant β-glucosidase process (baseline scenario)

In the baseline scenario, we estimate an annual enzyme production rate of 88 t of enzyme/year, as shown in Fig. 1. Considering an average sugarcane plant in Brazil that processes 2 million t of sugarcane/year, the annual production of β-glucosidase (BGL) proposed here would be sufficient to be used on the hydrolysis of approximately 39% of all the sugarcane bagasse produced annually. In this estimation, we assumed a bagasse/sugarcane fraction of 26% (w/w), a moisture content of 50%, a minimal BGL activity requirement of 0.2 CBU/FPU,^1^ an FPU activity requirement of 10 FPU/gDW of bagasse, and, at last, a BGL-specific activity of 2.3 CBU/mg of enzyme.

### Modeling and simulation software

SuperPro Designer v9.5 (Intelligen, USA) was employed to model and simulate both the baseline BGL production process and the variations of this process with respect to technical and economic parameters. The program was also used to perform an economic assessment of the process and to provide the input and output stream data necessary for the environmental assessment.

### Upstream section

Seed trains with expansion factors of 10-, 20-, and 100-fold (that is, 10, 5, and 1% of the inoculum volume, respectively) were evaluated. The expansion factor of 20 was used as a reference when evaluating parameters such as protein productivity. In all cases, the number of seed fermenters was determined by the initial effective (batch) volume of the main fermenter and the expansion factor chosen, considering that inoculum volumes of up to 20 L could be generated in a laboratory setting (not explicitly modeled). The medium used in the seed fermenters, as detailed in the next section, was the same in all cases.

### Fermentation section

#### Microorganism

*E. coli* BL21(DE3) harboring a pET28-a(+) plasmid (Novagen) carrying the *bglA* gene under control of the *lac* operator and T7 promoter was used as the expression system. The *blgA* gene codes for a thermostable, monomeric β-glucosidase, 52 kDa in size, were originally found in the hyperthermophilic, Gram-negative bacterium *Thermotoga petrophila* [26]. The plasmid also contains the kanamycin resistance gene *kanR*.

#### Culture media and fermentation conditions

The fermentation process was based on the fed-batch process proposed by Strittmatter et al. [27] and Horn et al. [28], which employs a defined medium containing glucose as the main carbon source and ammonia as the main nitrogen source. However, the replacement of glucose with glycerol was suggested by the original authors and was also evaluated. The entire process is carried out at 26 °C in a pressurized (150 kPa) stainless steel vessel, as summarized in Table 1. Initially, the microorganism consumes the substrates present in the batch medium (described in Additional file 1: Table S1), in which the carbon source (glucose or glycerol) is the only limiting substrate. When the carbon source concentration approaches a critical value (1.5 g/L), feeding solution 1 (FS1, also described in Table S1 of Additional file 1, together with feeding solutions 2 and 3) is added to maintain the carbon source concentration at a constant level. Therefore, the microbial culture process consists of a batch phase followed by a fed-batch phase. The control of glucose concentration, together with the use of a rather low growth temperature (26 °C) and a low-acetate-producing *E. coli* strain, such as BL21(DE3) [29], prevents the excessive production of acetate, thus allowing the bacteria to grow steadily at a constant specific growth rate, approximately 0.23 h^−1^, throughout the process.

Feeding solution 2 (FS2) and feeding solution 3 (FS3) are added at constant rates toward the end of the fed-batch process to avoid any contingent nitrogen or trace metal limitations. The volumes of FS2 and FS3 used in the simulations were proportional to the volume of the batch medium, following the ratios suggested by Horn et al. [28], i.e., 1 L of FS2 and 50 mL of FS3 for 8 L of batch medium. The proposed process also employs pH control, which is achieved by the addition of ammonium hydroxide (25% aqueous solution of NH_3_, w/w) to maintain a pH of 6.8. Ammonium hydroxide is particularly convenient because, as an additional benefit, ammonium hydroxide provides supplementary nitrogen to the cells. Since the production of organic acids was not considered in our model, the amount of base was calculated to precisely fulfill the nitrogen demand of the cells (which was not fulfilled by the batch medium and FS2).

Cell growth was modeled with stoichiometric equations based on the atomic balance of C, H, O, and N. The stoichiometric coefficient of the carbon source was set to 1 (on a molar basis), and the stoichiometric coefficient of the biomass was considered to be equal to the biomass yield from the carbon source. Values of 0.50 and 0.45 g/g of the biomass yield from the carbon source were assumed for the seed fermenters (batch) using glucose and glycerol, respectively. Biomass yields from glucose and glycerol during the main fermentation were assumed to be 0.40 and 0.36 g/g, respectively. The yield values for the seed (batch) fermentations are “theoretical” values found in Korz et al. [30]. The yield values for the main fermenter were estimated by applying a 20% reduction to the batch yield values. Based on our experience, the biomass yield can be reduced by as much as 50% in fed-batch mode compared to batch culture mode [31]. The glucose and glycerol values are both in reasonable agreement with those obtained by Wyre and Overton [32], who used somewhat similar conditions.

It was also assumed that the sole reactants in the growth equation were the main carbon source (glucose or glycerol), ammonia, and oxygen, whereas the only products were the *E. coli* biomass, with an empirical formula of CH_1.8_O_0.5_N_0.2_, according to the literature [33]; carbon dioxide; and water. These assumptions led to the stoichiometric equations used (presented in Table S2, Additional file 1). Additionally, in an alternative scenario, we considered that the enzyme was secreted to the broth, rather than stored inside the cell, as demonstrated by works in the literature [34]. The stoichiometric equation used in this case was derived from the equation of the baseline case, replacing a certain fraction of the dry cell weight by the enzyme.

In the baseline scenario, kanamycin sulfate was also added to the main fermenter and the seed fermenters at a final concentration of 30 mg/L. In the main fermenter, IPTG was also added to achieve final concentrations of 1 mM in the standard scenario and 0.1 mM in an alternative scenario, again assuming that everything else remained equal. Medium sterilization was performed in a continuous heat sterilizer. In contrast, thermo-sensitive compounds such as IPTG and kanamycin were assumed to be filter-sterilized in a laboratory (not explicitly modeled) and introduced directly into the fermenter. A compressor and an air filter were also included in the process model to generate a sterile air feed of 1 VVM. This flow rate was considered sufficient to maintain an oxygen pressure of 20%. Finally, an air filter was included downstream from the gas outlet of the fermenter to ensure the biosafety of the process. The fermentations in the seed fermenters were conducted in a similar fashion to the main fermentation batch phase, using the batch medium detailed in Table 1 as well as 30 mg/L of kanamycin and ammonium hydroxide for pH control and nitrogen supplementation; the fermentation process in each seed fermenter is described by the first two equations of Additional file 1: Table S2.

#### Productivity assumptions

Assuming that the specific growth rate remained constant and equal to 0.23 h^−1^ throughout the main culture and throughout the seed cultures, the duration of the main culture was calculated to be 19 h when the inoculum volume was 10%, 22 h when the volume was 5%, and 29 h when the volume was 1%. It should be noted that these calculations assumed that the seed fermenters employed the same batch medium previously described, that the seed fermenters had the biomass yields presented in Table 4, and that the biomass attained at the end of the main culture was 100 gDW/L. However, given the uncertainty associated with microbial cultures and with the expression of recombinant proteins, compounded with the uncertainty related to scaling up such processes, different scenarios of final biomass concentration and soluble rEnzyme content were evaluated, as described in Table S3 from Additional file 1. The highest value of the final biomass concentration, 120 g/L, was extracted from the graphical data of Horn et al. [28]. Lower values, 80 and 100 g/L, were also considered, since scaling up might diminish biomass yields, especially in the case of aerobic processes [28]. For the rEnzyme content, values of 2, 10, and 20% of the total protein, corresponding to 1, 5, and 10% of the dry cell weight, respectively, were evaluated. It should be stressed that except for the rEnzyme content, the overall cell composition was presumed to be the same in all cases, in accordance with the lysis equation presented later. The scenario with intermediate values of the final biomass and total rEnzyme content (Table S3, row #5, Additional file 1), i.e., 100 g/L and 5%, respectively, was assumed to be the standard scenario when comparing the different parameters. The alternative scenarios in which the enzyme was produced extracellularly assumed the same enzyme yield as that of intracellular production in the baseline case (Table S3, row #5, Additional file 1) or in the case of highest volumetric productivity (Table S3, row #9, Additional file 1).

### Downstream section

As the fermentation process ends, we assumed that the fermentation broth is collected in a tank and then passed through a high-pressure homogenizer, where the bacterial cells are lysed to harvest the intracellular protein product. Using the software, the homogenization process is modeled on a pseudo-reaction in which the biomass is converted into its main components. Based on the *E. coli* composition from Milo and Phillips [35], we developed the following (mass-based) lysis equation:

$$1 {\text{CH}}_{ 1. 8} {\text{O}}_{ 0. 5} {\text{N}}_{ 0. 2} \to 0.20\, {\text{Cell Debris}} + \left( {1 - \varphi } \right)\left( {0.50} \right)\, {\text{Contaminating Proteins}} + \varphi \left( {0.50} \right)\, {\text{rEnzyme }} + 0.20 \,{\text{DNA}} + 0.03 \,{\text{RNA }} + 0.03 \, {\text{Glycogen }} + 0.03 \,{\text{Metabolites }} + 0.01 \, {\text{Inorganic Ions}}.$$

In this equation, $\varphi$ is the rEnzyme content (rEnzyme/total protein) relative to the total soluble protein. The cell debris is then separated from the mixture by first using a disk-stack centrifuge, which removes 70% of the debris, and then with a dead-end filter, which removes the residual debris. Finally, the enzyme solution is concentrated to the desired titer (15 g of enzyme/L) and stabilized in a citrate buffer solution (citric acid + sodium citrate, pH 5.8) using a diafiltration system. The main parameters of the downstream operations are presented in Additional file 1: Table S4, and the complete process flowsheet of the baseline scenario is shown in Fig. 1. The main process sections—upstream, fermentation, and downstream—are indicated by different colors.

### Economic assessment

The economic assessment was performed using SuperPro Designer and was based on a plant located in the state of São Paulo, Brazil. The international cost of the equipment provided by SuperPro Designer software was used. Key cost data for the economic assessment and the corresponding sources of the data are provided in an additional file (Additional file 2: Tables S5 and S6).

## Results and discussion

Starting from the design parameters presented in the methodology section, we estimate an annual enzyme production rate of 88 t of enzyme/year in the baseline scenario, as shown in Fig. 1. As described previously (“Methods” section), we considered that the annual production of β-glucosidase proposed here would be sufficient to be used on the hydrolysis of approximately 39% of all the sugarcane bagasse produced annually by a Brazilian sugarcane plant that processes 2 million tons of sugarcane/year.

The process requires a seed train composed of two smaller fermenters, with volumes of approximately 0.2 and 4 m^3^. During each process cycle, 80 t of culture medium is fed to the main fermenter, and approximately the same amount of cell broth is found at the end of the microbial culture. This cell broth is collected in a storage tank and then lysed in a pressure homogenizer (throughput of 216 L/min). Next, the cell debris generated is removed using six disk-stack centrifuges (throughput of 36 L/min), and the remaining solids are removed by dead-end microfiltration (filter area of 50 m^2^). Finally, the enzyme solution is concentrated to 15 g/L and stabilized in a citrate buffer using a diafiltration system composed of eight ultrafilters (each with 74 m^2^ filter area) such that 22 tons of concentrated enzyme solution is produced per cycle, with a total of 264 cycles performed per year.

### Economic assessment

The overall unit production cost obtained for the baseline scenario was approximately 316 US$/kg of enzyme. This value is approximately 32 times higher than the estimated cost of the fungal enzyme mixture (10 US$/kg protein), as provided by Klein-Marcuschamer et al. [3], and also higher than other similar enzymes available in the literature (Additional file 3: Table S7). In the scenario proposed here, an on-site production of β-glucosidase for supplementation of fungal cellulases, such costly BGL would increase the final cost of the fungal cocktail by 137%. We estimated this value considering a fungal enzymatic cocktail with a specific filter-paper activity of 0.5 FPU/mg of protein, a BGL-specific activity of 2.3 CBU/mg, and that BGL is supplemented at a ratio of 0.2 CBU/FPU. This cost increase would not be justifiable in view of the observed effect of BGL supplementation on the enzymatic hydrolysis yield [10]. However, the baseline scenario was intentionally constructed based on conservative assumptions in order to better identify the advantages and limitations of the *E. coli* recombinant system in this context. Moreover, some simulated scenarios that are described in this work achieved substantially lower values of enzyme cost than the baseline scenario. Thus, in this section, we investigate all the simulated scenarios and the main drivers of enzyme cost with an eye toward possible cost reduction measures.

### Cost composition

**Fig. 2:**
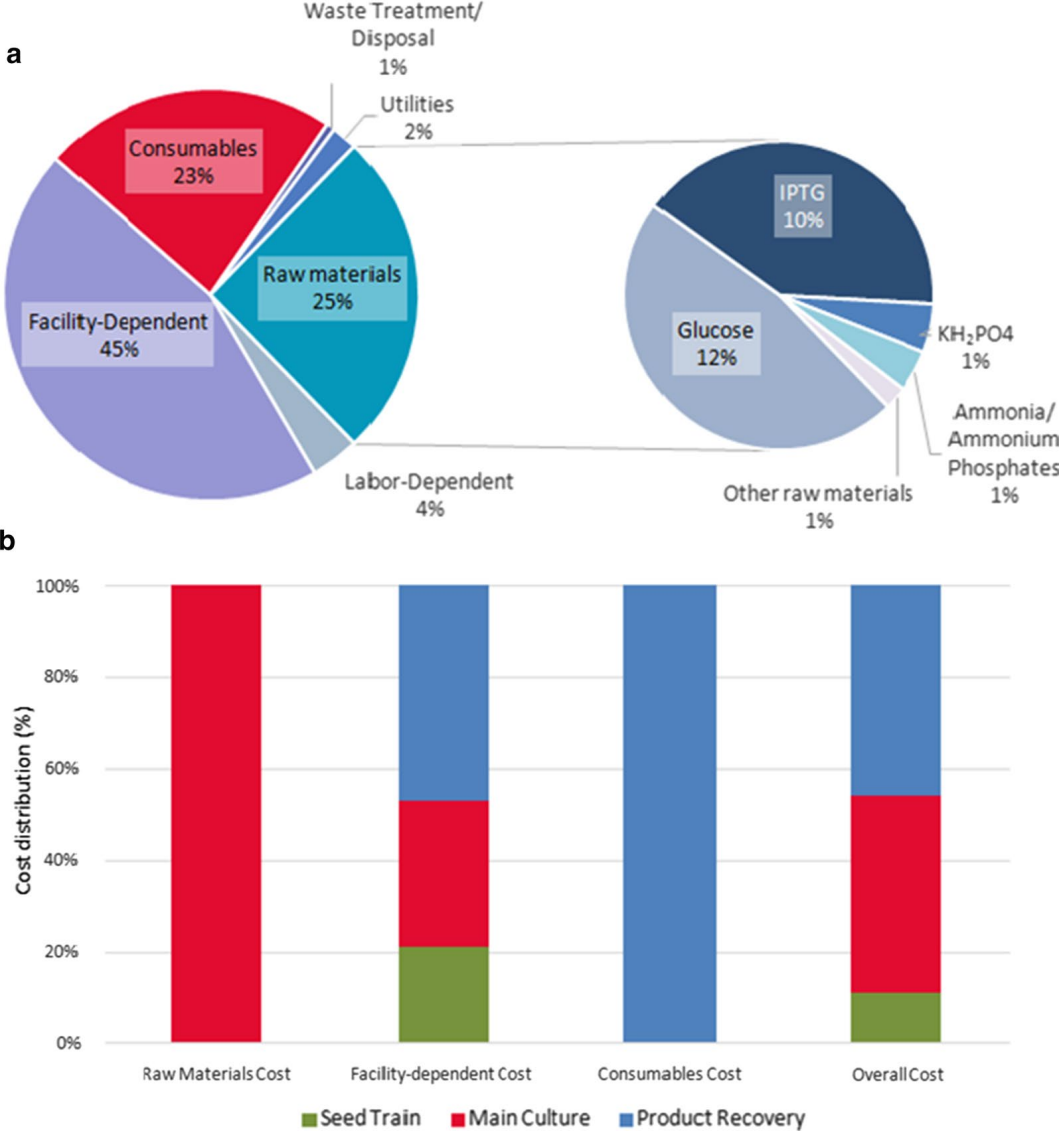
Cost composition. **a** Composition of the enzyme cost for recombinant β-glucosidase production using the baseline scenario. **b** Cost distribution across the different process sections of the production plant

Figure 2a shows that in the baseline case, facility-dependent costs, which include plant maintenance, depreciation, insurance, local taxes, and overhead costs not directly associated with the process (such as accounting, payroll, fire protection, and security), make up 45% of the unit production costs of the enzyme. Raw materials and consumables (filter cartridges and membranes) account for 25 and 23%, respectively. Since the facility-dependent cost is proportional to the purchased equipment cost in practice, as estimated by SuperPro Designer pricing models based on the US market, the facility-dependent cost may be somewhat overestimated. In fact, for example, Macrelli et al. [15] applied a factor of 0.82 to adjust the fixed capital cost of a bioethanol plant in the US Gulf Coast to Brazilian conditions. Given that the real/dollar currency exchange rate has increased dramatically in 2016 and that the proportion of equipment that would be imported is unknown, we decided not to apply any adjustment factor to the equipment cost provided by the software.

Of the costs of the raw materials, glucose and IPTG account for approximately 47 and 41%, respectively, and nitrogen- and phosphorus-rich compounds are together responsible for 10%. The costs of trace elements and, rather surprisingly, kanamycin seem to be negligible. These results confirm the common-sense idea that the use of less expensive carbon sources and induction strategies are important to reduce the enzyme cost.

Regarding the carbon source, it should be stressed that the cost of the glucose used was the market price of the compound. Therefore, it is reasonable to consider whether a glucose-rich liquor generated in the same (2G) plant could, at least in part, replace the purchased glucose, considerably reducing the cost of the carbon source. Similarly, one may envision replacing the purchased glucose with a glucose-fructose syrup obtained by inverting (hydrolyzing) sucrose in a 1G-plant setting or with glycerol, as suggested by Horn et al. [28]. The cost of glycerol, in particular, has decreased dramatically during the past decade, mainly because glycerol is a by-product of biodiesel production, which has greatly increased during the same period. Xylose-rich liquors generated from the hemicellulose hydrolysis process are also low-cost carbon sources that are not well utilized by the conventional ethanol-producing organism *S. cerevisiae*. Naturally, the use of these alternative and raw carbon sources could negatively impact the biomass and/or enzyme yields, since these carbon sources usually contain inhibitors of microbial metabolism.

Regarding the cost of induction, IPTG is widely considered to be too costly for the production of inexpensive recombinant proteins, especially at the concentrations at which IPTG is typically used in the laboratory (such as 1 mM). Our results confirm this perception. In fact, the cost contribution of IPTG is comparable to that of the main carbon source, which is 3 orders of magnitude less expensive. However, there are indications that lower IPTG concentrations may give rise to similar or sometimes better volumetric productivity of recombinant proteins, depending on the specific culture and expression conditions [32]. Since IPTG alone accounts for 10% of the unit production cost, reducing the amount of IPTG by one order of magnitude could, theoretically, reduce the enzyme cost by 9%. Alternative induction methods could also be explored, such as replacing IPTG with lactose (while keeping the *lac* operator) or employing a thermal induction system, which may be particularly convenient in cases in which the quantity and quality of the recombinant enzyme are not affected by this additional stress.

The effects of reducing the amount of IPTG (by tenfold), eliminating kanamycin from the process, and replacing glucose with glycerol were evaluated, assuming in the first two cases that biomass yield and protein productivity are unaffected. According to the simulations, kanamycin elimination and glycerol substitution make little difference in terms of cost, whereas IPTG reduction has a significant positive impact, reducing the enzyme cost by approximately 10%.

Additionally, the cost of consumables, which is quite significant (23%), is mainly due to the cost of the ultrafiltration membranes (80%) used in the diafiltration system and also the cost of the dead-end microfiltration cartridges (20%). Since the reason to use the dead-end filter is to avoid the fouling of the ultrafiltration membrane, one can conclude that, in our proposed process, the operation of the diafiltration unit has a large direct and indirect economic impact on the cost. In the pursuit of alternative units that are less expensive to operate, it might be interesting to concentrate the enzyme using different methods, such as by precipitation followed by centrifugation. However, the choice of the precipitation agent and the impact of this agent on enzyme activity, recovery yields, process complexity, and the environment should be experimentally evaluated. We have previously evaluated the potential of glycosyl hydrolase precipitation using ethanol under different temperature and pH conditions. In our experience, β-glucosidase activity can be almost fully recovered using 90% (v/v) ethanol at 25 °C and pH 6.5, indicating the potential of this solvent for use in a 2G ethanol plant [36].

The cost composition presented above refers to the overall process. However, this composition is far from uniform along the process, as seen in Fig. 2b. The facility-dependent cost, for example, is almost evenly distributed among the three main process sections, whereas the cost of raw materials is almost entirely due to the main fermenter feed, and the cost of consumables is entirely due to the downstream section (because the consumables are associated with the operation of filtration units). Overall, the fermentation section is the costliest section, followed by the downstream section. Nonetheless, the costs associated with the upstream section are significant (≈ 11%).

### Effects of scale and operating time

The presented enzyme costs were calculated considering a process scale that corresponds to a main fermenter volume of 100 m^3^. However, it is useful to evaluate the change in enzyme costs associated with a change in the scale of the process, especially considering that the percentage of bagasse set apart for 2G ethanol production in a plant would also depend on the relative prices of ethanol and electricity.

**Fig. 3:**
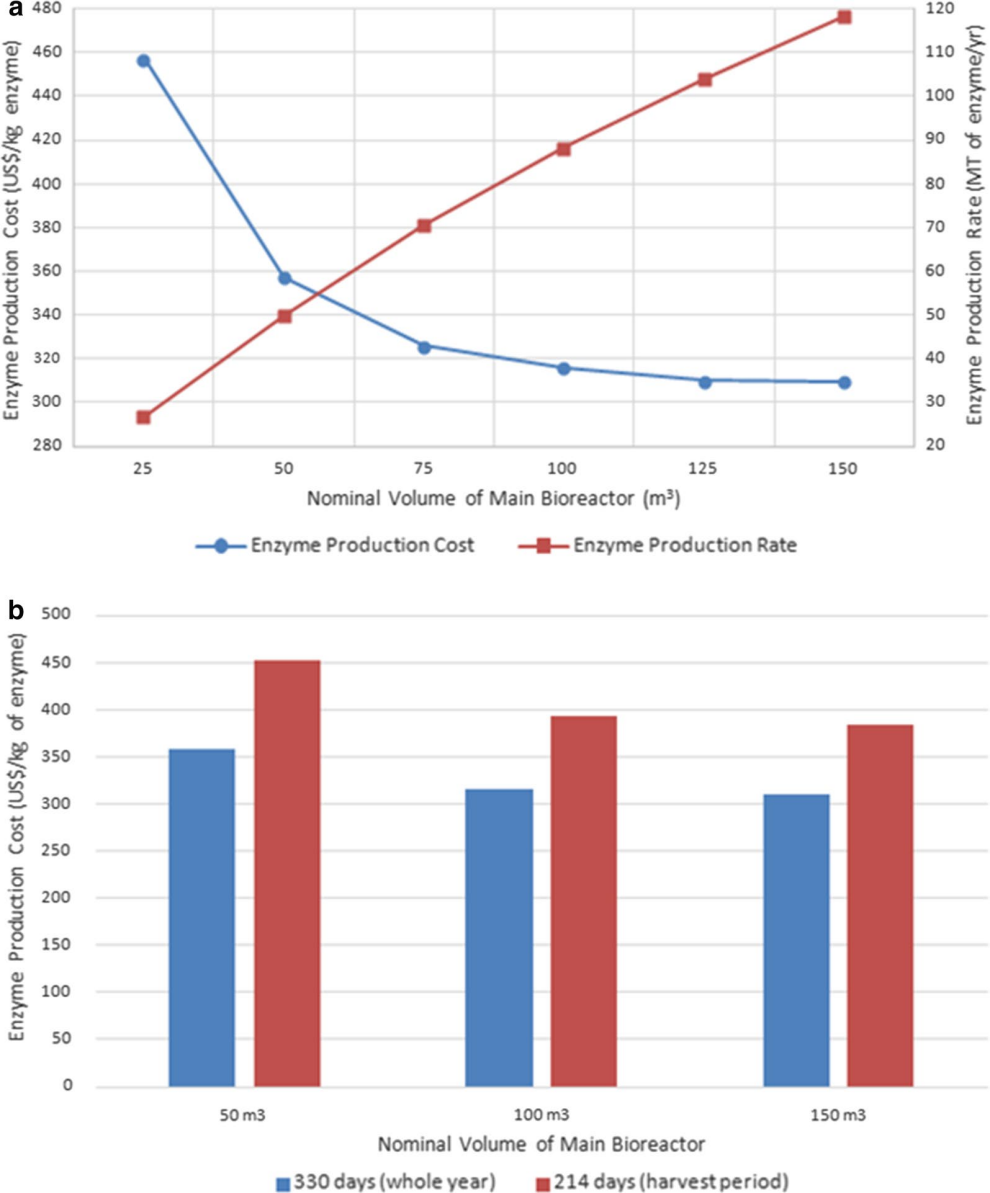
Effect of process scale and annual operating time on the baseline scenario. **a** Effect of process scale on enzyme cost and annual production rate. **b** Variation in enzyme cost with the annual operating time of the plant for different process scales

As shown in Fig. 3a, the amount of enzyme produced grows almost linearly with the scale of the process (represented by the main fermenter volume) throughout the range analyzed (from 25 to 150 m^3^). The unit production cost of the enzyme, in contrast, decreases in a non-linear manner as the process scale increases from 25 to 150 m^3^, decreasing drastically at the lower end of the scale and becoming almost flat at the higher end of the scale. The shape of this curve is typical of the phenomenon of economy of scale, largely because the facility-dependent cost becomes relatively smaller as the scale of the process increases. It should be mentioned, however, that the scale-down and scale-up of the process were performed using the software by simply adjusting the process throughput without considering any variations in biomass or enzyme yield that might arise from problems with oxygen transfer or other transport phenomena. Similar to the production scale, the annual operating time of the process strongly affects the unit production cost of the enzyme and is a particularly relevant parameter in the case of an on-site enzyme production plant dedicated to the hydrolysis of lignocellulosic biomass because the harvest of sugarcane does not occur throughout the calendar year but only between April and November, for approximately 7 months. Since sugarcane bagasse contains a high degree of moisture (50%), this material cannot be stored for long periods. In this context, Santos et al. [37] reported that natural bagasse loses approximately 30% of its calorific content in 150 days. Consequently, if the enzyme production unit were used only for bagasse hydrolysis and if there was no bagasse storage, the enzyme plant would remain idle for approximately 5 months/year [38].

Figure 3b shows how the enzyme cost increases, markedly, from approximately 316 to 393 US$/kg (for a 100 m^3^ fermenter), depending on the annual operating time of the plant. In contrast, if the moisture content of the biomass is decreased to approximately 20% or less, the biomass becomes essentially stable with respect to microbial activity [39, 40]. Therefore, it seems evident that a strategy for the safe, long-term storage of bagasse would have to be developed.

### Seed train

**Fig. 4:**
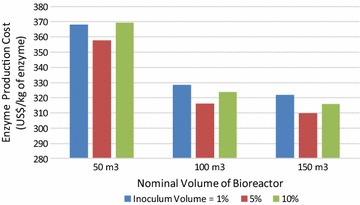
Effect of inoculum volume on enzyme cost for different process scales, with respect to the nominal volume of the bioreactor (fermenter)

Frequently overlooked in the literature, the seed train is a key part of any industrial bioprocess, since it is responsible for the propagation of the microorganism from small volumes to large bioreactors. Ideally, the seed train should preserve the desirable characteristics and the viability of the microorganism while avoiding any contamination. Here, the effect of inoculum volume on the cost of the recombinant enzyme for different process scales was simulated, and the result is presented in Fig. 4, as represented by the volumes of the main fermenter. For all three scales evaluated, an inoculum volume of 5% led to the lowest cost. At the lowest scale (50 m^3^), an inoculum volume of 10% led to the highest cost, whereas at the 100 and 150 m^3^ scales, an inoculum volume of 1% led to the highest cost. These results demonstrate the countervailing effects of inoculum size on enzyme cost: on one hand, larger inoculum volumes reduce the duration of the main culture, thereby increasing the number of batches per year and reducing the enzyme production cost. On the other hand, larger inoculum volumes require larger and more numerous seed bioreactors, thereby increasing the facility-dependent cost and the enzyme production cost. Either way, strategies to reuse a minor fraction of the main fermenter biomass instead of a seed train should be evaluated despite the potential decrease in productivity. In particular, the plasmid stability and transgene expression during long-term fermentation using *E. coli* would be critical for the production of low-cost enzymes. Hägg et al. [41] warned that the loss of plasmid vectors during bacterial cell division, leading to an increasing proportion of plasmid-free cells during growth, is a major industrial problem that results in reduced product yields and increased production costs during large-scale cultivation. It is worth noting that the seed train is often neglected in models of microbial processes.

### Fermentation section

**Fig. 5:**
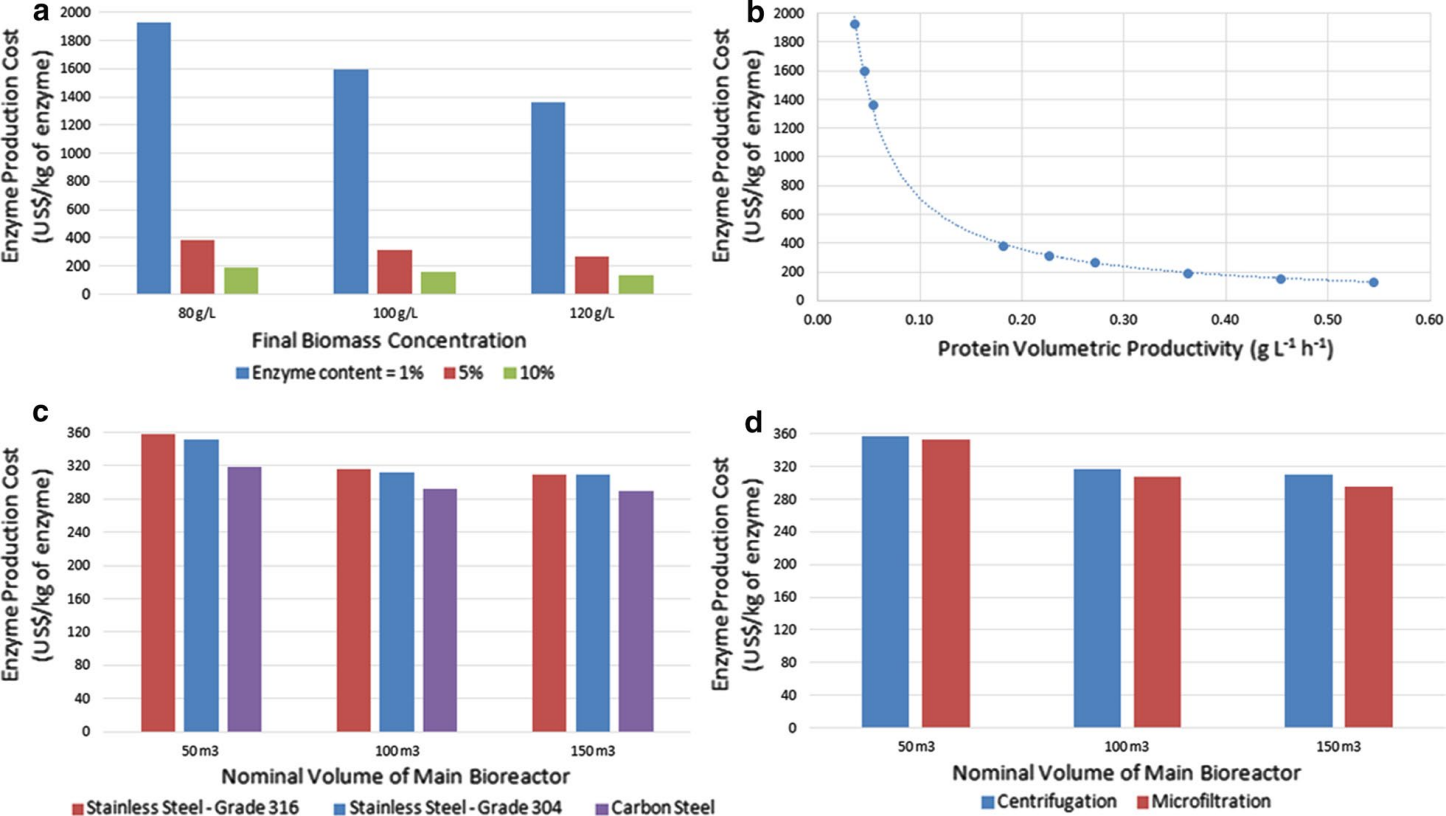
Effects of enzyme volumetric productivity, bioreactor material, and cell clarification method on the final enzyme production cost. **a** Variation in enzyme cost with the final biomass concentration and the rEnzyme content (w/w). **b** Variation in enzyme cost with enzyme volumetric productivity. **c** Effect of bioreactor material on final enzyme production cost. Labels: SS316, stainless steel of grade 316; SS304, stainless steel of grade 304; and CS, carbon steel. **d** Comparison of downstream operations for cell debris separation and the impact of the final enzyme cost at three different process scales

The effect of the biomass concentration at the end of the fermentation and the effect of the recombinant protein (rEnzyme) content are presented in Fig. 5a. Clearly, both variables have a dramatic influence on enzyme cost; the case where the lowest biomass is coupled with the lowest rEnzyme content (1926 US$/kg) and the case where the highest biomass is coupled with the highest rEnzyme content (135 US$/kg) are 14 orders of magnitude apart. These results confirm the importance that has generally been ascribed to the volumetric productivity of fermentation processes, which is defined as the mass of the product at the end of the process divided by the final broth volume and the duration of fermentation. To better visualize the effect of this parameter, the biomass concentration and rEnzyme content data were combined and converted into volumetric productivity and plotted against enzyme cost, as shown in Fig. 5b. The chart shows that the enzyme cost does indeed decrease rapidly with volumetric productivity, and the data are very well approximated by a power law in which the cost is inversely proportional to the volumetric productivity. However, it is well known that rEnzyme content may negatively correlate with biomass concentration as a result of the so-called metabolic burden of recombinant protein synthesis and as a result of the toxic properties of inducer molecules such as IPTG [28]. Consequently, these findings indicate the need for a better understanding of the tradeoffs involved in recombinant protein production and, in particular, for experimentally identifying optimum induction conditions and the final biomass concentration range.

A parameter frequently overlooked in techno-economic analyses of bioprocesses is the material of the fermenter, which must resist the frequently corrosive products and by-products of fermentation. Grade 316 stainless steel is considered to be the standard bioreactor material for most biotechnological processes [42]. In fact, grade 316 steel was the material used in the simulations of fungal cocktail production performed by Humbird et al. [2]. However, the authors proposed the use of a lower grade of stainless steel, 304, for a *Zymomonas* fermenter. Furthermore, carbon steel has a precedent in the American corn ethanol industry [2] and the Brazilian sugarcane ethanol industry [43]. Thus, simulations were conducted assuming that the fermenter and seed fermenters were made of stainless steel of higher grade (SS316), stainless steel of lower grade (SS304), or a carbon-steel alloy (CS); the simulations were conducted using cost models from SuperPro Designer. The results are shown in Fig. 5c. The use of SS304 had a negligible effect on the enzyme cost, whereas the substitution of carbon steel for stainless steel was somewhat significant, decreasing the cost from 316 to 292 US$/kg on a process scale of 100 m^3^. However, these results do not account for possible requirements for corrosion prevention, such as the application of special coatings [43] or the need to design a thicker fermenter [2]. Therefore, the replacement of SS316 with less expensive materials is not warranted by these results.

### Downstream processing

Despite being extremely streamlined, the downstream section accounts for nearly half of the enzyme cost, as discussed above. This can be partially attributed to the contribution of the downstream section to the facility-dependent cost (43% of the total). At this point, the cytoplasmic production of proteins by *E. coli* introduces a significant, but not critical, increase in costs.

The replacement of centrifugation by microfiltration was also evaluated. The choice between these unit operations is commonly encountered in the biotechnology industry, and although centrifugation is generally considered to be more cost effective at smaller scales [44], the choice must be evaluated on a case-by-case basis. The results presented in Fig. 5d indicate that centrifugation is the best option to separate cell debris at both the baseline scale (100 m^3^) and the smaller (50 m^3^) scale, whereas microfiltration was less costly at the larger scale (150 m^3^). Nevertheless, the difference in cost between these two separation methods was negligible at the smaller scale and quite modest (approximately 5%) at the largest scale.

Alternative downstream operations, such as precipitation using ethanol, might be used to concentrate enzymes from the clarified lysate [45]. However, additional unit operations for precipitate recovery (centrifugation or filtration) and resuspension have to be considered. Aqueous-two phase systems (ATPS) have also been effectively used for separation and purification of industrial proteins. An elegant review on this topic was published by Ansejo and Andrews [46]. The production and purification of chymosin from recombinant *Aspergillus* supernatant is the most successful industrial application of this technology. Silvério et al. [47] studied the separation and purification of laccase from a complex fermented medium using an ATPS system with a thermo-separating polymer. Despite the possible recovery and reutilization of the polymer, a large loss of activity was observed (88%) when compared with the classical PEG-Salt systems. In general, precipitation and ATPS are adequate if some increase in the purity level of the protein is needed [33]. Although a more comprehensive study of the possible downstream process designs would be very interesting, here we have focused on the most common downstream process configurations described in similar studies, such as those listed in the Additional file 3: Table S7 [3, 5, 48].

Finally, an important factor that can be attributed to the low cost of fungal cellulases is the simplification of the downstream process as a result of the extracellular secretion of the enzymes. The use of cheaper unit operations for cell removal, such as the vacuum drum filter (well suited to fungal biomass separation), may also contribute to the reduced fungal enzyme cost. Although we have no information on β-glucosidase secretion by *E. coli*, it was interesting to simulate scenarios in which this enzyme could be secreted at the same expression levels as those assumed for intracellular expression. In these cases, centrifugation was employed to separate the cells from the liquid phase containing the enzyme. The impact of enzyme secretion on cost reduction was confirmed because of the simplification of the downstream process (Table S8, Additional file: 3). Moreover, the cost of the enzyme was strongly dependent on the solids content of the sludge. Considering a solid content of 580 g/L (~ 24% of dry cell weight) in the sludge, an enzyme cost reduction of 9% was achieved in relation to the baseline scenario.

### Searching for optimized processes

On the search for a better understanding of the factors contributing to the technical and economic feasibility of the process under study, we generated a comprehensive set of simulated scenarios of recombinant BGL production, by varying many of the process characteristics and parameters discussed so far, one by one. The results are compiled in Additional file 3: Table S8, which shows the individual impact on enzyme cost (relatively to the baseline scenario) from each parameter. Additional simulated scenarios combining changes in several parameters and process conditions can also be seen in Additional file 3: Table S8.

As expected, enzyme titer presented the most important impact on cost. The reduction of the cost of glucose, the reduction of the amount of IPTG added, and the use of less expensive bioreactor materials (carbon steel) also proved important (11, 9, and 10% of cost reduction, respectively) (rows #2, and 4). A large cost reduction was also achieved when combining the factors presented above and eliminating unit operations used to concentrate and stabilize the enzyme, thus strengthening the decision of on-site production of enzymes in the context of biorefineries. In this case, a production cost of 63 US$/kg of enzyme was found (row #15). Finally, the last (and best) scenario presented in Table S8 shows that enzyme secretion, combined with the previous efforts to optimize the protein production process, could ultimately reduce the enzyme cost to 37 US$/kg (row #25). Although still higher than the values found in some published studies for fungal cellulases, this value indicates that, with improvements on the enzyme expression level and adequate choices in process design, enzyme costs in the range of 40–70 US$/kg could be reached using recombinant *E. coli*.

## Conclusions

Despite the considerable technical and economic uncertainties that surround 2G ethanol and the large-scale production of low-cost recombinant enzymes in high-cell-density cultures, we believe that our model sheds light on some relevant questions. First, facility-dependent costs pose a significant challenge for the production of low-cost, high-volume enzymes by microorganisms in general and specifically by *E. coli*. Second, considering our baseline scenario, the cost of recombinant enzyme production in *E. coli* (316 US$/kg) is high in relation to the average cost of fungal cellulase cocktails suggested by the literature. However, the results also indicate that the final enzyme cost could be reduced on many fronts, e.g., by replacing the carbon source with cheaper alternatives, changing the induction strategy, or improving the inoculation process and volumetric productivity. The combination of the optimized upstream scenario with a very streamlined downstream section, thanks to the use of an *E. coli* strain able to secrete the target enzyme, would ultimately lead to a final cost of 37 US$/kg of protein.

Altogether, this work provides a comprehensive analysis of the factors affecting the cost associated with the production of low-value enzymes using *E. coli* on a large scale, contributing to a better understanding of the actual potential use of this host in industry, especially in the context of tailor-made enzymatic cocktails for 2G ethanol production.

## Additional files

**Additional file 1.** Main process parameters and assumptions. This file details the composition of the growth media, the stoichiometric equations used to model microbial growth, the main parameters of the main culture process, as well as the main parameters of the downstream section.

**Additional file 2.** Cost data and economic indices. This file lists the costs of raw materials, utilities, labor, financing, as well as price indices used in the economic analysis.

**Additional file 3.** Detailed simulation data—optimized scenarios. This file lists a compilation of the results for cellulase production found in the literature, including results of this work. There is also a list of 25 different simulation scenarios for the recombinant β-glucosidase process generated in this work.

## Abbreviations

1G [ethanol/plant]: first generation [ethanol/plant]
2G [ethanol/plant]: first generation [ethanol/plant]
rEnzyme/rProtein: recombinant enzyme/recombinant protein
FS1/2/3: feeding solution 1/2/3
BGL: β-glucosidase
CBU: cellobiase activity unit
FPU: filter-paper activity unit

## Footnotes

1 CBU: cellobiase activity unit; FPU: Filter-paper activity unit.
