# Supplementary material for: Techno-economic analysis of the industrial production of a low-cost enzyme using E. coli: the case of recombinant β-glucosidase
Source: Biotechnol Biofuels. 2018 Mar 29;11:81. doi: 10.1186/s13068-018-1077-0 (PMC5875018; doi:10.1186/s13068-018-1077-0)
Supplement: Supplementary file 1 — Additional file 1. Main process parameters and assumptions. This file details the composition of the growth media, the stoichiometric equations used to model microbial growth, the main parameters of the main culture process, as well as the main parameters of the downstream section. [file 13068_2018_1077_MOESM1_ESM.docx]

**Additional file 1: Main Process Parameters and Assumptions**

This file details the composition of the growth media, the stoichiometric equations used to model microbial growth, the main parameters of the main culture process as well as the main parameters of the downstream section.

Table S1: Culture media used in the rEnzyme production process.

| Component | Batch Medium (mg/L) | Feed Solution 1 (mg/L) | Feed Solution 2 (mg/L) | Feed Solution 3 (mg/L) | Others |
| --- | --- | --- | --- | --- | --- |
| Glucose  (Glycerol) | 25 × 10^3^ (27.8 × 10^3^) | 670 × 10^3^ (744.4 × 10^3^) |  |  |  |
| MgSO_4_× 7H_2_O | 1.5 × 10^3^ | 19.8 × 10^3^ |  |  |  |
| K_2_HPO_4_ | 16.6 × 10^3^ |  |  |  |  |
| Citric acid | 2.1 × 10^3^ |  |  |  |  |
| (NH_4_)_2_HPO_4_ | 4 × 10^3^ |  | 227 × 10^3^ |  |  |
| (NH_4_)H_2_PO_4_ |  |  | 169.5 × 10^3^ |  |  |
| Fe(III) citrate hydrate | 75 |  |  | 5 × 10^3^ |  |
| H_3_BO_3_ | 3.8 |  |  | 250 |  |
| MnCl_2_× 4H_2_O | 18.8 |  |  | 125 |  |
| EDTA × 2H_2_O | 10.5 |  |  | 700 |  |
| CuCl_2_× 2H_2_O | 1.9 |  |  | 125 |  |
| Na_2_MO_4_× 2H_2_O | 3.1 |  |  | 213 |  |
| CoCl_2_× 6H_2_O | 3.1 |  |  | 213 |  |
| Zn(CH_3_COO)_2_× 2H_2_O | 10 |  |  | 668 |  |
| Aqueous NH_3_ (NH_4_OH) |  |  |  |  | 25% w/w |
| Kanamycin Sulfate |  |  |  |  | 30 mg/L* |
| IPTG |  |  |  |  | 1 mmol/L* |

* Concentration given with respect to the solution volume; that is, 30 mg of kanamycin is added to every liter of fermentation medium.

Table S2: Stoichiometric equations to describe cell growth on either glucose or glycerol; these coefficients are mass-based.

| Seed Fermenters |
| --- |
| Glucose:  $\boldsymbol{180}\boldsymbol{.}\boldsymbol{16} \text{C}_{\boldsymbol{6}}\text{H}_{\text{12}}\text{O}_{\text{6}}\boldsymbol{+}\boldsymbol{69}\boldsymbol{.}\boldsymbol{10}\text{ O}_{\text{2}}\boldsymbol{+}\boldsymbol{12}\boldsymbol{.}\boldsymbol{46} \text{N}\text{H}_{\text{3}}\boldsymbol{\to}\boldsymbol{90}\boldsymbol{.}\boldsymbol{08} \text{C}\text{H}_{\text{1,8}}\text{O}_{\text{0,5}}\text{N}_{\text{0,2}}\boldsymbol{+}\boldsymbol{103}\boldsymbol{.}\boldsymbol{08} \text{C}\text{O}_{\text{2}}\boldsymbol{+}\boldsymbol{68}\boldsymbol{.}\boldsymbol{55} \text{H}_{\text{2}}\text{O}$ |
| Glycerol:  $\boldsymbol{92}\boldsymbol{.}\boldsymbol{09} \text{C}_{\boldsymbol{3}}\text{H}_{\text{8}}\text{O}_{\text{3}}\boldsymbol{+}\boldsymbol{55}\boldsymbol{.}\boldsymbol{45}\text{ O}_{\text{2}}\boldsymbol{+}\boldsymbol{5}\boldsymbol{.}\boldsymbol{73} \text{N}\text{H}_{\text{3}}\boldsymbol{\to}\boldsymbol{41}\boldsymbol{.}\boldsymbol{44} \text{C}\text{H}_{\text{1,8}}\text{O}_{\text{0,5}}\text{N}_{\text{0,2}}\boldsymbol{+}\boldsymbol{57}\boldsymbol{.}\boldsymbol{96} \text{C}\text{O}_{\text{2}}\boldsymbol{+}\boldsymbol{53}\boldsymbol{.}\boldsymbol{87} \text{H}_{\text{2}}\text{O}$ |
| Main Fermenter |
| Glucose:  $\boldsymbol{180}\boldsymbol{.}\boldsymbol{16} \text{C}_{\boldsymbol{6}}\text{H}_{\text{12}}\text{O}_{\text{6}}\boldsymbol{+}\boldsymbol{93}\boldsymbol{.}\boldsymbol{67}\text{ O}_{\text{2}}\boldsymbol{+}\boldsymbol{9}\boldsymbol{.}\boldsymbol{97} \text{N}\text{H}_{\text{3}}\boldsymbol{\to}\boldsymbol{72}\boldsymbol{.}\boldsymbol{06} \text{C}\text{H}_{\text{1,8}}\text{O}_{\text{0,5}}\text{N}_{\text{0,2}}\boldsymbol{+}\boldsymbol{135}\boldsymbol{.}\boldsymbol{27} \text{C}\text{O}_{\text{2}}\boldsymbol{+}\boldsymbol{76}\boldsymbol{.}\boldsymbol{46} \text{H}_{\text{2}}\text{O}$ |
| Glycerol:  $\boldsymbol{92}\boldsymbol{.}\boldsymbol{09} \text{C}_{\boldsymbol{3}}\text{H}_{\text{8}}\text{O}_{\text{3}}\boldsymbol{+}\boldsymbol{66}\boldsymbol{.}\boldsymbol{76}\text{ O}_{\text{2}}\boldsymbol{+}\boldsymbol{4}\boldsymbol{.}\boldsymbol{59} \text{N}\text{H}_{\text{3}}\boldsymbol{\to}\boldsymbol{33}\boldsymbol{.}\boldsymbol{15} \text{C}\text{H}_{\text{1,8}}\text{O}_{\text{0,5}}\text{N}_{\text{0,2}}\boldsymbol{+}\boldsymbol{72}\boldsymbol{.}\boldsymbol{78} \text{C}\text{O}_{\text{2}}\boldsymbol{+}\boldsymbol{57}\boldsymbol{.}\boldsymbol{51} \text{H}_{\text{2}}\text{O}$ |

Table S3: Values of biomass and rEnzyme content.

| Scenario | Culture Duration (h) | Biomass  (gDCW L^-1^) | rEnzyme/ Total Protein | rEnzyme/ Biomass | rEnzyme Volumetric Productivity (g L^-1^ h^-1^) |
| --- | --- | --- | --- | --- | --- |
| 1 | 22 | 80 | 2% | 1% | 0.04 |
| 2 | 22 | 80 | 10% | 5% | 0.18 |
| 3 | 22 | 80 | 20% | 10% | 0.36 |
| 4 | 22 | 100 | 2% | 1% | 0.05 |
| 5 | 22 | 100 | 10% | 5% | 0.23 |
| 6 | 22 | 100 | 20% | 10% | 0.45 |
| 7 | 22 | 120 | 2% | 1% | 0.05 |
| 8 | 22 | 120 | 10% | 5% | 0.27 |
| 9 | 22 | 120 | 20% | 10% | 0.54 |

Table S4: Main Downstream Parameters.

| Parameter | | Value |
| --- | --- | --- |
| Homogenization | | |
| Number of Passes | | 2 |
| Pressure | | 1000 kPa |
| Cell Disruption | | 100% |
| rEnzyme Denaturation | | 5% |
| Centrifugation | | |
| Sedimentation Efficiency | | 30% |
| Cell Debris Removal | | 70% |
| Solid Concentration in Heavy Stream | | 200 g/L |
| Microfiltration | | |
| Whole-Cell Rejection Coefficient | | 1.00 |
| Cell Debris Rejection Coefficient | | 0.95 |
| rEnzyme Denaturation | | 3% |
| Concentration Factor | | 10 |
| Dead-End Filtration | | |
| Cell Debris Removal | | 100% |
| Contaminant Protein Removal | | 5% |
| Nucleic Acid Removal | | 5% |
| rEnzyme Removal |  | 1% |
| Particle Concentration in Retentate (v/v) | | 50% |
| Diafiltration | | |
| Volume Permeated | | 1 |
| Contaminant-Protein Rejection Coefficient | | 0.80 |
| rEnzyme Rejection Coefficient | | 1.00 |
| rEnzyme Denaturation | | 3% |
| Concentration Factor | | variable* |

*Factor chosen so that the final rEnzyme concentration was equal to 15 g/L.
