# Supplementary material for: Techno-economic analysis of the industrial production of a low-cost enzyme using E. coli: the case of recombinant β-glucosidase
Source: Biotechnol Biofuels. 2018 Mar 29;11:81. doi: 10.1186/s13068-018-1077-0 (PMC5875018; doi:10.1186/s13068-018-1077-0)
Supplement: Supplementary file 2 — Additional file 2. Cost data and economic indices. This file lists the costs of raw materials, utilities, labor, financing, as well as price indices used in the economic analysis. [file 13068_2018_1077_MOESM2_ESM.docx]

**Additional file 2: Cost Data and Economic Indices**

This file lists the costs of raw materials, utilities, labor, financing as well as price indices used in the economic analysis.

Table S5: Cost of raw materials. Except where indicated, the prices of all raw materials were market prices obtained at the Molbase Chemical E-Commerce platform ([www.molbase.com](http://www.molbase.com), accessed May 2017).

| Raw Material | Price (US$/kg) |
| --- | --- |
| Glycerol | 0.61 |
| Glucose | 0.66 |
| MAP | 0.349^a^ |
| Ammonia Gas | 0.303^b^ |
| DAP | 0.353^b^ |
| KH_2_PO_4_ | 1.29 |
| Process Water | 0.00018^c^ |
| Citric Acid | 0.82 |
| CoCl_2_ | 9.52 |
| CuCl_2_ | 4.30 |
| EDTA*Na_2_ | 2.74 |
| H_3_BO_3_ | 0.73 |
| Iron III Citrate | 7.63 |
| MgSO_4_ | 0.48 |
| MnCl2 | 1.71 |
| Na_2_MoO4 | 13.48 |
| Zn(OAc)_2_ | 1.72 |
| Kanamycin Sulfate | 31.72 |
| IPTG | 601.00 |

^a^ Mosaic Corporation (2016); ^b^ Potachcorp (2016); ^c^ Macrelli et al. (2012).

Table S6: Costs of utilities, labor and financing.

| Parameter | Value | Unit | Source |
| --- | --- | --- | --- |
| Utilities |  |  |  |
| Cooling Water (20°C) | 0.04 | US$/t | Macrelli et al. (2012) |
| Chilled Water (5°C) | 0.35 | US$/t | SuperPro default |
| Steam | 1.47 | US$/t | Mussatto et al. (2013) |
| Electricity | 56.00 | US$/(kW.h) | Brazilian Federal Government, (2016) |
| Waste Treatment |  |  |  |
| Filter Cake | 4.07 | US$/t | Theodoro (2005) |
| Aqueous Waste | 1.71 | US$/m^3^ | Theodoro (2005) |
| Labor cost |  |  |  |
| Basic Rate | 7.93 | US$/h | US Bureau of Labor Statistics (2016) |
| Operating Supplies | 10% |  | SuperPro default |
| Supervision | 20% |  | SuperPro default |
| Administration | 60% |  | SuperPro default |
| Adjusted Rate | 15.07 | US$/h | Calculated from above |
| Time Parameters |  |  |  |
| Month/Year of Analysis and Construction | May 2016 |  | Defined |
| Construction Period | 30 | months | Defined |
| Startup Period | 6 | months | Defined |
| Project Lifetime | 25 | years | Defined |
| Financing |  |  |  |
| Debt | 50% of project |  | BNDES, Brazil (2016) |
| Loan Period | 6 | years | BNDES, Brazil (2016) |
| Loan Interest Rate | 10.25% |  | Calculated BNDES rate for medium/large ethanol plants |
| Price Indices |  | |  |
| Chemical Engineering Plant Cost Index (CEPCI) | Piping/Equipment Estimated using SuperPro Design | | Chemical Engineering Magazine (2016) |
| Nominal Average Salary in Sao Paulo state | Labor cost in Brazilian Plant | | IPEA, Brazil (2016) |
| Unit labor cost data for the Chemical Industry | Update of American Labor Cost | | US Bureau of Labor Statistics (2016) |
| Producer Price Index (USA) | Utilities Cost Update | | US Bureau of Labor Statistics (2016) |
| Producer Price Index (Brazil) - Processing/Oil/Biofuels Industry | Utilities and Materials from Brazilian Sources | | IPEA, Brazil (2016) |
| IPA-M | Update of Waste Treatment Costs in Brazil | | IPEA, Brazil (2016) |
| Dollar/Real Exchange Rate | 3.54 | | Brazilian Central Bank (2016) |

**References**

Macrelli S, Mogensen J and Zacchi G. Techno-economic evaluation of 2nd generation bioethanol production from sugar cane bagasse and leaves integrated with the sugar-based ethanol process. Biotechnol Biofuels. 2012;5:22.

Mussatto SI, Moncada J, Roberto IC, Cardona CA. Techno-economic analysis for brewer's spent grains use on a biorefinery concept: The Brazilian case. BioresTechnol. 2013;148, 302:310.

Mosaic Corporation. Brazil NPK Statistical Update - May 2016. Available from: http://www.mosaicco.com/documents/Brazil_NPK_Update___May_2016_Statistics.pdf. Accessed 15 May 2016.

PotashCorp. Market Data - Selected Fertilizer Prices. Available from: http://www.potashcorp.com/customers/markets/market_data/prices/. Accessed 14 May 2016.

Theodoro JMP. Considerações sobre os Custos Ambientais Decorrentes do Gerenciamento dos Resíduos Sólidos e dos Efluentes Industriais Gerados no Setor Sucroalcooleiro: Um Estudo de Caso [Internet]. Centro Universitário de Araraquara; 2005. Available from: http://www.uniara.com.br/mestrado_drma/arquivos/dissertacao/Jose_Marcos_Paula_Theodoro_2005.pdf. Accessed 15 May 2016.
