# Supplementary material for: Techno-economic analysis of the industrial production of a low-cost enzyme using E. coli: the case of recombinant β-glucosidase
Source: Biotechnol Biofuels. 2018 Mar 29;11:81. doi: 10.1186/s13068-018-1077-0 (PMC5875018; doi:10.1186/s13068-018-1077-0)
Supplement: Supplementary file 3 — Additional file 3. Detailed simulation data—optimized scenarios. This file lists a compilation of the results for cellulase production found in the literature, including results of this work. There is also a list of 25 different simulation scenarios for the recombinant β-glucosidase process generated in this work. [file 13068_2018_1077_MOESM3_ESM.docx]

**Additional file 3**

This file lists a compilation of the results for cellulase production found in the literature, including results of this work. There is also a list of 25 different simulation scenarios for the recombinant β-glucosidase process generated in this work.

Table S7: Economic data of cellulase production process.

| Expression system/Enzyme | Production mode | Enzyme cost ($/Kg) | Enzyme production rate  (MT/year) | AOC  ($ Mi) | CC  ($ Mi) | Facility-dependent cost (%) | Reference |
| --- | --- | --- | --- | --- | --- | --- | --- |
| *Trichoderma reesei* cellulase | Submerged fermentation | 10.0 | 2,820.0 | 29.0 | 76.9 | 48.0 | [3] |
| *Clostridium thermocellum* cellulase | Submerged fermentation | 40.0 | 757.0 | 30.5 | 28.6 | 22.0 | [48] |
|  | Semi-solid state fermentation | 15.0 | 525.0 | 8.2 | 22.0 | 63.0 |  |
| Transgenic Tobacco cellulase | High density field cultivation | 9.0 | 2,870.0 | 20.0 | 11.5 | Not considered | [5] |
| Recombinant *E. coli* β-glucosidase | High cell density cultivation | 316.0 | 88 | 27.9 | 70.8 | 43.0 | This work |
|  |  | 37.0 | 286.0 | 10.7 | 47.0 | 64.0 | This work |

AOC – Annual operating cost; CC – Capital cost

Table S8: Scenarios of a recombinant β-glucosidase process production generated in this work.

|  | Enzyme expression | Enzyme titer^a^ (g/L) | Bioreactor material | Kanamicyn | IPTG amount (g) | Glucose cost  ($) | Cell /  Debris removal | Enzyme recovery/  Concentration | Sludge/ Cake concentration (g of solids/L) | Additional bioreactor^b^ | Final enzyme titer^c^  (g/L) | Enzyme cost  ($/kg) | Cost Reduction |
| --- | --- | --- | --- | --- | --- | --- | --- | --- | --- | --- | --- | --- | --- |
| 1 | Intracellular | 5 | SS316 | yes | 1X | 1X | CT | DE+UF+DF | 200 | no | 15.0 | 316 | - |
| 2 | Intracellular | 5 | CS | yes | 1X | 1X | CT | DE+UF+DF | 200 | no | 15.0 | 283 | 10% |
| 3 | Intracellular | 5 | SS316 | yes | 0.1X | 1X | CT | DE+UF+DF | 200 | no | 15.0 | 286 | 9% |
| 4 | Intracellular | 5 | SS316 | yes | 1X | 0.1X | CT | DE+UF+DF | 200 | no | 15.0 | 282 | 11% |
| 5 | Intracellular | 5 | SS316 | yes | 1X | 1X | MF | DE+UF+DF | 200 | no | 15.0 | 349 | -10%^d^ |
| 6 | Intracellular | 5 | SS316 | yes | 1X | 1X | CT | DE+UF | 200 | no | 15.0 | 292 | 8% |
| 7 | Intracellular | 5 | SS316 | yes | 1X | 1X | CT | DE+UF+DF | 200 | yes | 15.0 | 296 | 6% |
| 8 | Intracellular | 12 | SS316 | yes | 1X | 1X | CT | DE+UF+DF | 200 | no | 15.0 | 135 | 57% |
| 9 | Intracellular | 12 | SS316 | yes | 1X | 1X | CT | no | 200 | no | 11.5 | 92 | 71% |
| 10 | Intracellular | 12 | CS | yes | 1X | 1X | CT | no | 200 | no | 11.5 | 78 | 75% |
| 11 | Intracellular | 12 | SS316 | no | 1X | 1X | CT | no | 200 | no | 11.5 | 92 | 71% |
| 12 | Intracellular | 12 | SS316 | yes | 0.1X | 1X | CT | no | 200 | no | 11.5 | 79 | 75% |
| 13 | Intracellular | 12 | SS316 | yes | 1X | 0.1X | CT | no | 200 | no | 11.5 | 75 | 76% |
| 14 | Intracellular | 12 | SS316 | yes | 1X | 1X | MF | no | 200 | no | 11.2 | 101 | 68% |
| 15 | Intracellular | 12 | SS316 | yes | 1X | 1X | CT | no | 200 | yes | 11.5 | 86 | 73% |
| 16 | Intracellular | 12 | SS316 | yes | 0.1X | 0.1X | CT | no | 200 | no | 11.5 | 63 | 80% |
| 17 | Extracellular | 5 | SS316 | yes | 1X | 1X | CT | DE+UF+DF | 580 | no | 15.0 | 289 | 9% |
| 18 | Extracellular | 12 | SS316 | yes | 1X | 1X | CT | no | 580 | no | 18.4 | 85 | 73% |
| 19 | Extracellular | 12 | CS | yes | 1X | 1X | CT | no | 580 | no | 18.4 | 72 | 77% |
| 20 | Extracellular | 12 | SS316 | no | 1X | 1X | CT | no | 580 | no | 18.4 | 85 | 73% |
| 21 | Extracellular | 12 | SS316 | yes | .1X | 1X | CT | no | 580 | no | 18.4 | 73 | 77% |
| 22 | Extracellular | 12 | SS316 | yes | 1X | .1X | CT | no | 580 | no | 18.4 | 68 | 78% |
| 23 | Extracellular | 12 | SS316 | yes | 0.1X | 0.1X | CT | no | 580 | no | 18.4 | 56 | 82% |
| 24 | Extracellular | 12 | SS316 | yes | 1X | 1X | MF | no | 580 | no | 17.9 | 92 | 71% |
| 25 | Extracellular | 12 | SS316 | yes | 0.1X | 0.1X | CT | no | 580 | yes | 18.4 | 37 | 88% |

CT: Centrifugation; DE: Dead-End Filtration; DF: Diafiltration (with ultrafiltration membrane); MF: Microfiltration; UF: Ultrafiltration; CS: Carbon steel; SS316: Stainless

steel of grade 316.

^a^ After cell disruption (in the case of intracellular expression) or cell harvesting (in the case of extracellular expression).

^b^ Additional bioreactor: equipment to be used in stagger mode with the main bioreactor.

^c^ After primary recovery unit operations.

^d^ Enzyme cost increases by 10%.
